# The common prosperity effect of rural households' financial participation: a perspective based on multidimensional relative poverty

**DOI:** 10.3389/fpubh.2024.1457921

**Published:** 2024-12-10

**Authors:** Zhengyin Li, Xiangdong Hu

**Affiliations:** Institute of Agricultural Economics and Development, Chinese Academy of Agricultural Sciences, Beijing, China

**Keywords:** formal financial participation, informal financial participation, common prosperity, multidimensional relative poverty, land transfer, non-farm employment

## Abstract

**Introduction:**

Common prosperity holds significant importance in ensuring social equity, promoting sustainable economic growth, and achieving long-term national security. The management of multidimensional relative poverty is a crucial pathway to realizing the common prosperity of all individuals. It is worthwhile to investigate whether the formal and informal financial involvement of rural households can synergistically alleviate multidimensional relative poverty, ultimately contributing to the realization of common prosperity.

**Methods:**

Using data from 5,303 farm households in the 2018 China Family Panel Studies, this study employs multiple linear regression, instrumental variable methods, and propensity score matching to empirically analyze the common prosperity effect of formal and informal financial participation from the perspective of multidimensional relative poverty.

**Results:**

The research demonstrates that both formal and informal financial participation can alleviate multidimensional relative poverty, with formal financial participation exhibiting a more pronounced poverty reduction effect compared to informal financial participation. Mechanism analysis reveals that both forms of financial participation mitigate multidimensional relative poverty by facilitating land transfer and non-farm employment. Heterogeneity analysis reveals that formal financial participation yields a more pronounced poverty reduction effect among rural households experiencing lower levels of multidimensional relative poverty, whereas informal financial participation is more effective in reducing poverty among rural households facing higher levels of multidimensional relative poverty.

**Discussion:**

To achieve common prosperity and enhance the precision of financial interventions for poverty alleviation, it is recommended to leverage the strengths of formal finance over informal finance, enhance financial assistance for land transfer and non-farm employment, and implement tailored financial support policies.

## 1 Introduction

Common prosperity holds significant importance in ensuring social equity, promoting sustainable economic growth, and achieving long-term national stability. The theoretical connotation of common prosperity encompasses affluence and equitable distribution. Overcoming poverty serves as a fundamental prerequisite for attaining affluence, while mitigating relative disparities stands as a crucial aspect of fostering equitable distribution. Consequently, common prosperity manifests at the micro level through the reduction of multidimensional relative poverty (1). In 2020, China reached a significant milestone in its battle against poverty by historically eliminating absolute poverty, demonstrating remarkable achievements in poverty alleviation efforts. However, the eradication of absolute poverty does not imply the cessation of poverty alleviation efforts (2), as the challenge of relative poverty will persist indefinitely. China's rural regions are focal points of relative poverty, with rural households constituting the largest vulnerable demographic in the country. These households are susceptible to reverting to poverty when confronted with risks such as natural disasters and disease outbreaks, posing challenges to the realization of common prosperity. Consequently, assisting rural households in breaking free from multidimensional relative poverty is imperative to meeting the populace's aspirations for improved living standards and advancing common prosperity.

Finance has historically played a crucial role in governing rural poverty (3). Formal financial services in rural areas can address capital shortages among rural households by providing credit, enhance household income through savings and financial management, and mitigate production and operational risks through insurance. As a result, these initiatives contribute to improving household income and fostering economic development in impoverished regions. However, the effectiveness of rural formal finance in reducing poverty remains limited. For instance, despite government efforts to support formal financial lending to rural households through subsidies and other means in the financial poverty alleviation process, rural formal finance has been unable to fully meet rural households' credit needs due to factors such as the absence of collateral (4). Consequently, the expansion of rural households' production and operations encounters financing constraints, impeding the demonstration of the poverty reduction effect and hindering the achievement of common prosperity. Rural informal finance, leveraging its unique information channels, low transaction costs, and flexible collateral methods, has expanded rural households' access to financing. It has effectively addressed the challenge of obtaining loans for rural households and mitigated the limitations of rural formal finance. In numerous regions, informal finance has emerged as the primary source of funding for rural households, establishing a complementary dynamic with formal finance (5). Consequently, can formal and informal finance work synergistically to reduce poverty? This study aims to investigate the mechanisms and heterogeneity of formal and informal rural finance in reducing multidimensional relative poverty. The findings provide a foundation for designing financial poverty alleviation policies and offer theoretical support for tailoring financial assistance to different types of rural households. Ultimately, this research seeks to promote the synergy between formal and informal finance in reducing multidimensional relative poverty and contribute to the achievement of common prosperity.

## 2 Literature review

### 2.1 Research on multidimensional relative poverty

Current research on multidimensional relative poverty focuses on three key areas. First, the conceptual evolution of multidimensional relative poverty. Poverty studies have gradually shifted from absolute and relative poverty to multidimensional and multidimensional relative poverty. Initially, absolute poverty was defined as a state where a household's total income could not cover the basic necessities required by all its members to maintain normal bodily functions (6). With a deeper understanding of poverty, scholars argued that it also includes relative exclusion and deprivation, leading to the development of the relative poverty theory (7). However, relative poverty has its limitations (8), necessitating consideration of various factors that affect the long-term wellbeing of impoverished individuals, such as health, education, and quality of life (9). Consequently, some scholars argue for the establishment of a multidimensional poverty measurement system to address the comprehensive development needs of the poor (10). While multidimensional relative poverty is distinct from multidimensional poverty, the concept of relative poverty emphasizes differences among individuals. Its measurement dimensions and indicators cannot be directly equated with those of multidimensional poverty. Furthermore, existing research on multidimensional relative poverty often adopts the measurement dimensions of multidimensional poverty without adequately addressing the distinctions in measurement elements (11). Multidimensional relative poverty is a dynamic form of poverty characterized by a state of living in which a family's health, education, and standard of living are significantly below the socially recognized average due to factors such as uneven economic development and disparities in resource endowments (12). Second, the measurement of multidimensional relative poverty. Unlike absolute poverty, relative poverty was initially measured based on farm household income using relative income indicators. For example, the European Union uses 60% of median disposable income per capita as the threshold for relative poverty, while other studies have adopted varying benchmarks, such as 50% of average income (13), 60% of median income (14, 15), or 40% of disposable income (16). Although low income is a key factor contributing to poverty, it is not the only determinant of farmers' wellbeing (17), making it necessary to consider multiple dimensions. Alkire et al. proposed the “double-boundary method” to measure multidimensional poverty (18). Building on this approach, scholars have developed multidimensional relative poverty indices across various dimensions, tested the robustness of these methods (19, 20), and applied them to analyze multidimensional relative poverty among different groups (21). Third, the factors influencing multidimensional relative poverty. Key factors that significantly affect the multidimensional relative poverty of rural households include livelihood capital and type (22), rural social pension insurance (23), risk attitudes (24), mobile payments (25), the rural digital economy (26), Internet access (27), labor mobility (28), land transfers (29), and government transfer payments (30).

### 2.2 Finance and poverty alleviation

The existing literature on the relationship between finance and poverty can be broadly divided into two perspectives: one advocates for the pivotal role of finance in poverty alleviation. Viewed from a macro standpoint, financial development stimulates regional economic growth, affording the impoverished access to development benefits, thereby mitigating rural poverty and decreasing overall poverty rates (31, 32). Moreover, the progression of financial development has spurred financial institutions to extend their presence into rural regions, fostering rural economic growth and reducing the urban-rural income disparity (33). Viewed from a micro standpoint, finance plays a beneficial role for households, resulting in poverty alleviation (34). For instance, savings services allow individuals to earn interest income and manage consumption during periods of low income. Similarly, credit services help mitigate financial constraints, facilitate the accumulation of human and physical capital, and decrease poverty rates (35). The development of inclusive finance has enabled poor households to access credit, not only raising their income levels but also improving their wellbeing in various dimensions, such as health and education (36). Another perspective contends that finance exacerbates poverty. At the macro level, financial development exacerbates income inequality, widening income disparities and increasing relative poverty through financial exclusion and elite capture (37, 38). On a micro level, financial development facilitates easier access to credit and financial products, prompting households to borrow. However, high interest rates, default risks, and income volatility hinder households from effectively managing debt, thereby contributing to increased poverty (39). Additionally, financial development offers an array of financial choices and investment opportunities, but without adequate financial literacy and guidance, households might engage in high-risk investments or illicit financial activities (40), resulting in financial losses and exacerbating poverty.

### 2.3 Literature commentary

In summary, the existing literature reveals the complexity of multidimensional relative poverty and the necessity of its multidimensional measurement, while discussing the relationship between financial development and poverty. Although this literature provides a rich theoretical foundation for a deeper exploration of the link between finance and poverty, several gaps remain. First, most studies have analyzed the impact of financial development on poverty from the supply side, with few addressing the participation of rural households in both formal and informal finance from the demand side, making it difficult to comprehensively assess their combined effects on impoverished rural households. Second, there is insufficient investigation into the mechanisms through which these two forms of financial participation influence the multidimensional relative poverty of rural households. Third, further refinement of multidimensional relative poverty is needed to explore how formal and informal financial participation affects households with varying dimensions and levels of poverty, thereby optimizing the poverty reduction potential of financial participation. To address these gaps, This paper introduces three key innovations: first, novel research perspective and analytical framework: unlike prior studies that primarily examine the impact of financial development on poverty from the supply side (3, 5), this study adopts a demand-side perspective. It integrates both formal and informal financial participation by farmers into a unified analytical framework, examining their individual and synergistic effects on reducing multidimensional relative poverty. This comprehensive approach addresses the limitations of existing literature, which often focuses exclusively on a single type of financial participation, thereby offering a more holistic perspective. Second, in-depth analysis of poverty reduction mechanisms: existing literature largely focuses on macro-level mechanisms linking financial development to poverty reduction, with limited exploration of specific pathways (4). This paper conducts a systematic mechanism analysis to examine how formal and informal financial participation reduce multidimensional relative poverty by promoting farm households' economic activities, such as land transfer and non-farm employment. By emphasizing micro-level pathways, this study offers more targeted insights for policymakers. Third, decomposition and heterogeneity analysis of multidimensional poverty: unlike traditional studies that treat poverty as a single-dimensional measure (9, 10), this paper decomposes multidimensional relative poverty into three dimensions: earning capacity, development capacity, and living environment. It examines the varying impacts of financial participation on each dimension of poverty. Additionally, the paper employs quantile regression to analyze the heterogeneity of poverty reduction effects among farm households at different poverty levels. This refined analytical framework enhances the breadth and depth of multidimensional poverty research.

## 3 Theoretical analysis and research hypotheses

### 3.1 The influence of formal and informal financial participation by rural households on multidimensional relative poverty

According to Lin et al., formal financial participation of rural households is defined as households' adherence to legitimate, standardized, and formal channels such as banks, credit cooperatives, and cooperatives, under the regulation of laws, to engage in financial activities such as savings, credit, and insurance (41). The objective is capital allocation, risk management, and wealth appreciation. In contrast, informal financial participation of rural households refers to their reliance on informal channels like relatives, friends, and mutual aid associations to compensate for their exclusion from formal finance.

Financial participation among rural households can mitigate multidimensional relative poverty. Specifically, savings services furnish rural households with avenues to save and accrue interest earnings, thereby creating supplementary funds for future investments and expenditures. Credit services can not only provide farmers with start-up capital or help expand the scale of their existing production operations, thus increasing the output and quality of their products and boosting their incomes through technological improvements and the acquisition of machinery and equipment, but also assist in enhancing the education levels of both themselves and their children, as well as improving their housing conditions. Insurance services allow rural households to receive compensation in the event of risks such as natural disasters, diseases, and market price fluctuations. This helps alleviate their financial burden, mitigate business risks, and safeguard their incomes and assets.

In this study, we utilize the research conducted by Tan et al. as a reference point regarding loans and enhance it to investigate the impacts of formal and informal financial engagement among rural households on multidimensional relative poverty (42). In a perfectly competitive market scenario, let there be “*m*” rational rural households, each requiring a loan “*C*” to initiate a business project. The interest rate for the loan is denoted by “*r*, ” and the return function for each project is represented by “*R*(*C*).” The average probability of project success is “*p*, ” yielding a net return of “*R*(*C*)−*C*(1+*r*)” upon success for rural households. Conversely, the average probability of project failure is “1−*p*, ” resulting in the repayment of both the principal and interest rate, with a net return of “−*C*(1+*r*).”

Assuming that rural households are restricted to borrowing solely from formal financial institutions, owing to the phenomena of financial exclusion and elite capture, only “*a*” rural households can secure loans from such institutions at an interest rate of “*r*_*z*_.” At this point, the expected returns for rural households are:


(1) $$\begin{array}{r} {\text{Y}}_{1}=\text{ap}\left[\text{R}\left(\text{C}\right)-\text{C}\left(1+{\text{r}}_{\text{z}}\right)\right]+\text{a}\left(1-\text{p}\right)\left[-\text{C}\left(1+{\text{r}}_{\text{z}}\right)\right]\\ =\text{a}\left[\text{pR}\left(\text{C}\right)-\text{C}\left(1+{\text{r}}_{\text{z}}\right)\right] \end{array}$$

When a rural household fails to secure a loan, the expected return is 0. Hence, there exists a critical lending rate that enables formal financial participation to augment the return for the rural household, denoted as:


(2) $$\begin{array}{l} {\text{r}}_{\text{z}}\leq \frac{\text{pR}\left(\text{C}\right)}{\text{C}}-1 \end{array}$$

Rural households unable to secure loans from formal financial institutions start seeking funds from informal financial sources. Assuming that an additional “b” rural households obtain informal financial loans at an interest rate of “*r*_*f*_, ” the expected return for all rural households becomes:


(3) $$\begin{array}{r} Y_{2}=ap\left[R\left(C\right)-C\left(1+r_{z}\right)\right]+a\left(1-p\right)\left[-C\left(1+r_{z}\right)\right]+bp[R\left(C\right)\\ -C\left(1+r_{f})\right]+b\left(1-p\right)[-C\left(1+r_{f})\right]\; \end{array}$$

The anticipated yield for a rural household that solely obtains an informal financial loan is:


(4) $$\begin{array}{r} Y_{2}-Y_{1}=bp\left[R\left(C\right)-C\left(1+r_{f}\right)\right]+b\left(1-p\right)\left[-C\left(1+r_{f}\right)\right]\\ =b\left[pR\left(C\right)-C\left(1+r_{f}\right)\right] \end{array}$$

At this juncture, there exists a critical lending rate that enables informal financial participation to augment the returns to rural households, i.e:


(5) $$\begin{array}{l} {\text{r}}_{\text{f}}\leq \frac{\text{pR}\left(\text{C}\right)}{\text{C}}-1 \end{array}$$

When formal and informal finance meet the critical lending rate conditions outlined in Equations 2, 5 respectively, and this requirement must be fulfilled under the assumption of a perfectly competitive market and rational actors, both formal and informal financial participation can enhance the returns of rural households. Moreover, informal financial participation can further augment the returns of rural households based on the enhancements brought about by formal financial participation. Based on the preceding analysis, this study posits research hypothesis H1.

H1. Both formal and informal financial engagement by rural households can alleviate multidimensional relative poverty, with informal financial participation building on formal financial engagement to further alleviate poverty.

### 3.2 Pathways through which the formal and informal financial participation of rural households influence multidimensional relative poverty

Expanding the scope of agricultural operations and engaging in non-farm employment are pivotal strategies to augment rural household income. The capacity to generate income serves as the cornerstone for enhancing development capabilities and ameliorating living conditions within rural communities. It directly mirrors the economic vigor and sustainability potential of rural households. Consequently, this study focuses on land transfer and non-farm employment as conduits through which rural household financial engagement impacts multidimensional relative poverty.

Formal and informal financial participation can facilitate land transfer and off-farm employment for rural households. Land transfer encompasses both inflow and outflow of land. For land inflows in land transfers, when rural households acquire land from others, they must pay land rent, and expanding land holdings requires hiring labor and purchasing machinery and equipment. However, financial constraints hinder poor rural households from effectively utilizing land resources and expanding agricultural production, thereby impeding land acquisition. Formal or informal credit services can address the financing needs of these households, bridging the financial gap for land rent payment and enabling the acquisition of high-quality machinery and equipment. This, in turn, enhances the marginal return on land and incentivizes land acquisition. For land outflows in land transfers, Formal and informal finance support rural households' entrepreneurial activities during land outflows in land transfers, leading to a notable rise in non-farm labor and a significant reduction in agricultural labor, thereby facilitating land outflows. Non-farm employment includes entrepreneurship or working outside the home. Non-farm employment encompasses entrepreneurship or working outside the home. For entrepreneurial activities in off-farm employment of rural households, the presence of financial constraints hampers poor rural households from accessing adequate start-up and operational capital for entrepreneurial endeavors in off-farm employment. This limitation curtails the scale and progress of entrepreneurial initiatives, thereby constraining their competitiveness and access to market opportunities. Formal and informal credit services can provide the necessary initial capital for impoverished rural households to initiate their own businesses, fostering an improved entrepreneurial environment and facilitating the expansion of project scope for these households. Regarding rural households' non-farm employment, when they seek work beyond their homes, they must possess the requisite knowledge and labor skills to contend with the intense competition and diverse job demands in the market. However, impoverished rural households struggle to make sustained investments in their human capital, limiting their employment prospects largely to low-skilled manual labor. This not only renders them easily replaceable but also disincentivizes their engagement in off-farm work. Through engagement in formal or informal finance, impoverished rural households can secure funds for education and training, fostering the enhancement of their educational attainment and professional skills. Consequently, this amplifies their human capital and competitiveness within the labor market, enabling them to attain stable non-farm employment.

Land transfer and non-farm employment can alleviate the multidimensional relative poverty experienced by rural households. Firstly, land inflow in land transfer can facilitate the large-scale operation of agricultural land. Through the adoption of advanced mechanical equipment and technology, this process harnesses economies of scale, significantly enhancing land use efficiency and labor productivity. Moreover, it encourages rural households to transition toward cultivating higher-value-added cash crops, consequently elevating their income levels (43). Land outflow in land transfer not only boosts rural household income through transfer fees but also frees up surplus labor within the household. This surplus labor can then seek non-farm employment opportunities in urban areas, leading to increased wage incomes (44). Secondly, entrepreneurial endeavors in non-agricultural sectors not only offer job prospects for rural households themselves but also attract employment from neighboring rural residents. This helps curtail the unemployment rate, broaden employment horizons, augment the incomes of impoverished rural households, and mitigate rural poverty. Engaging in non-agricultural employment provides rural households with supplementary income sources, aiding impoverished rural communities in bolstering their earnings and enhancing living standards, and rural households have the opportunity to access diverse vocational training and skill enhancement programs, thereby enhancing their employability and competitiveness, thus increasing their chances of securing better job prospects and higher incomes. Thirdly, land transfer and non-farm employment not only increase the income of rural households but also introduce them to new social roles, exposing them to diverse experiences and concepts within economic and social contexts. This exposure stimulates and strengthens their developmental sense, prompting them to prioritize endogenous development capabilities such as health, children's education, and social security. Additionally, they develop higher expectations for their living environments. As a result, there is a significant reduction in the extent of multidimensional relative poverty. Based on the preceding analysis, this study posits research hypothesis H2.

H2. Formal and informal financial engagement among rural households plays a crucial role in facilitating land transfer and off-farm employment, thereby contributing to the alleviation of multidimensional relative poverty.

### 3.3 The varying effects of formal and informal financial participation on rural households across different levels of poverty

Formal finance relies on a comprehensive credit system and is characterized by high credit limits but also high service thresholds. In contrast, informal finance relies on familial and social relationships and is characterized by lower credit limits but flexible operations, simple and efficient procedures, and lower service thresholds. These differences in characteristics result in distinct target audiences for formal and informal financial services among rural households, leading to varying effects on poverty reduction across different rural households.

Formal financial institutions need to ensure sufficient capital and control risks to ensure robust operations and provide safe and reliable financial services. This necessitates a higher entry threshold for formal financial services. As a result, rural households with lower levels of multidimensional relative poverty may meet these requirements to access formal financial services, thereby reducing multidimensional relative poverty. On the one hand, formal financial institutions typically offer higher credit limits, enabling them to provide financing support on a larger scale. Rural households with lower levels of multidimensional relative poverty often possess stronger economic capacities and better credit histories. As a result, formal financial institutions are more inclined to extend higher credit limits to these households. This facilitates the expansion of their operations and investment in new technologies and equipment, leading to faster and more sustainable poverty reduction. On the other hand, Formal financial institutions typically provide an extensive array of financial products and services, spanning areas such as deposits, loans, insurance, and wealth management. This breadth of offerings empowers rural households experiencing low levels of multidimensional relative poverty to address their financial needs more comprehensively. For instance, they can opt to open savings accounts for fund accumulation, access loans to enhance production capacities, and procure insurance to mitigate risks. These financial instruments play a crucial role in bolstering the economic activities and advancement of rural households, fostering income growth, and more effectively fulfilling their poverty alleviation mandate. Additionally, rural households experiencing lower levels of multidimensional relative poverty are more inclined to alleviate this poverty by establishing a favorable credit history through punctual repayments and credit accumulation. This enables them to access increased financial support and more favorable lending terms, enhancing their flexibility and sustainability in future economic endeavors. In conclusion, formal financial engagement proves to be more efficacious in poverty alleviation for rural households with lower levels of multidimensional relative poverty.

Informal financial institutions, characterized by flexible operational models, streamlined application processes, and minimal transaction costs, have reduced the barriers to accessing financial services for rural households with lower economic capacity and smaller financial requirements. On one hand, informal financial institutions exhibit greater flexibility in addressing the financial requirements of rural households, featuring lower service thresholds and simplified procedural demands. Rural households grappling with significant levels of multidimensional relative poverty often encounter obstacles in securing loans, stemming from poor credit histories, unstable income sources, or failure to meet the criteria of formal financial institutions. In contrast, informal finance leverages familial and social networks, circumventing the need for rural households to fulfill extensive documentation and reference requirements. This streamlined approach facilitates easier access to financial assistance, enabling rural households to address their daily living and economic development needs. On the other hand, rural households experiencing high levels of multidimensional relative poverty contend with various poverty indicators simultaneously, including educational deficits, low income, and medical challenges. Faced with resource constraints, they must prioritize their expenditures. Access to informal financial services enables them to allocate funds toward addressing poverty in areas where costs are lower, facilitating the reduction of multidimensional relative poverty. In conclusion, informal financial participation proves more effective in alleviating poverty among rural households experiencing higher levels of multidimensional relative poverty. Based on the preceding analysis, this study posits research hypothesis H3.

H3. Formal financial participation proves more effective in alleviating poverty among rural households experiencing lower levels of multidimensional relative poverty, whereas informal financial participation is more effective for those facing higher levels of multidimensional relative poverty.

## 4 Variable selection and model specification

### 4.1 Data sources

This study utilizes survey data from the 2018 China Family Panel Studies (CFPS) as its subject of analysis. The household questionnaire of the CFPS contains information about the household's basic demographics, residential area, socioeconomic activities, among other factors. Meanwhile, the individual questionnaire captures individual characteristics such as health status, education level, and economic situation. Thus, the CFPS2018 questionnaire data offers robust support for this study. In this study, the sample data underwent sequential screening and processing as follows: aligning individual and household data; retaining samples of rural household heads; excluding samples with household heads under 18 or over 70 years old; and excluding rural households with total cash and deposits exceeding 1 million yuan. Following these steps, a dataset comprising 5,303 rural household samples was obtained.

### 4.2 Variable definitions and descriptive statistics

(1) Dependent variables: Multidimensional relative poverty

Common prosperity is reflected at the micro level as a reduction in multidimensional relative poverty (1); thus, the explanatory variable in this study is multidimensional relative poverty. In order to achieve this objective, this study constructs a multidimensional relative poverty indicator system for rural households, drawing on multidimensional poverty measurement theory and the multidimensional poverty index from the Human Development Report. Additionally, referring to Dong et al. and Wang et al., the multidimensional relative poverty indicator system for rural households shown in Table 1 is constructed based on the data from the 2018 China Family Panel Studies (45, 46). The indicator encompasses three dimensions, eight indicators, and 13 questionnaire items. In terms of income capability, this study adopts a methodology similar to that of Sun et al. to define a rural household as income-capability poor if its per capita disposable income is below 50% of the median per capita disposable income among all sampled rural households (16). Regarding development capability, the study concentrates on the health status, education level, and social security level of rural households. And on the living environment dimension, this study not only examines the housing area, consumer goods, and living standards of rural households but also integrates subjective attitudes into the indicator system.

**Table 1:** Multidimensional relative poverty indicator system for rural households.

**Dimension**	**Indicators**	**Meaning of indicators**	**Indicator interpretation and assignment**
Income capacity (1/3)	Income (1/3)	Net per capita income	Assigning a value of 1 to households with net per capita income below 50% of the median, and otherwise 0
Development capacity (1/3)	Health (1/9)	Self-assessed health status of adults, number of medical consultations for children	Adults who self-report as “unhealthy” or children who have had more than four medical consultations in a year in the household are assigned a value of 1, otherwise 0
	Education (1/9)	Adults' years of education, school-age children's enrolment status	Adults with < 6 years of schooling on average or children of school age not attending school in the household are assigned a value of 1, otherwise 0
	Social security (1/9)	medical insurance enrolment	A value of 1 is assigned if there is an employed individual in the household without any health insurance coverage; otherwise 0
Living environment (1/3)	Housing (1/12)	Housing ownership, per capita housing area	Households with either no property or < 15 m^2^ of housing per capita are assigned a value of 1; otherwise 0
	Consumer goods (1/12)	Value of consumer durables	The household is assigned a value of 1 if the total worth of consumer durables owned is < 1,000 yuan; otherwise 0
	Living standard (1/12)	Water for cooking, fuel for cooking	The type of cooking water is not classified as “well water,” “tap water,” “purified water,” or the fuel used is “firewood” or “straw,” it is assigned a value of 1; otherwise 0
	Subjective attitude (1/12)	Life satisfaction, personal social status	Indicates “very dissatisfied” or “very low social status” are assigned a value of 1, otherwise 0

In constructing the multidimensional poverty index, this paper assigns equal weights to each dimension and indicator, grounded in the principles of fairness, practicality, and the minimization of subjective bias. First, from the perspective of fairness, equal weighting assumes that all dimensions and indicators contribute equally to multidimensional relative poverty, enabling the index system to comprehensively reflect the poverty status of farmers. Sen's capability approach underscores the equal importance of multiple dimensions to individual wellbeing, making equal weighting an extension of this theory that captures the collective influence of all dimensions on poverty (9). Second, from a practical standpoint, the multidimensional relative poverty index aims to create an accessible and operational system for policy analysis and socioeconomic research. Using equal weights avoids overly complex calculations, resulting in a concise and straightforward system that policymakers can readily understand and apply. Third, from the perspective of reducing subjective bias, assigning different weights in a multidimensional relative poverty measure may lead to varied conclusions, particularly among interrelated and significant dimensions such as social security, health, and education. Therefore, using equal weights helps minimize bias and avoids disputes over weight selection. Furthermore, this approach aligns with the Global Multidimensional Poverty Index developed by the United Nations Development Program and the Oxford Poverty and Human Development Initiative, as well as related studies (16). The use of equal weights ensures a balanced assessment across dimensions of poverty. For studying rural poverty in China, equal weighting provides a comprehensive depiction of farm households' poverty status across all dimensions, rather than focusing narrowly on specific domains.

(2) Core Independent Variables: Formal and informal financial participation

The main independent variables in this study consist of dummy variables representing formal and informal financial participation. Specifically, formal financial participation is indicated by a value of 1 for a rural household that receives loans from formal financial institutions or holds bank deposits, and 0 otherwise. Informal financial participation is indicated by a value of 1 for a rural household that obtains loans from other organizations or individuals, or borrows money from individuals or organizations, and 0 otherwise.

(3) Control variables

This study incorporates control variables at individual, household, and societal levels. At the individual level, factors such as the gender, age, political affiliation, and marital status of the household head are considered. Household-level variables include household size, per capita savings, and per capita expenditure on favors. At the societal level, government subsidies and social donations are taken into account. Other control variables are better understood. Expenditures on favors refer to spending on gifts, gratuities, and other non-essential items by rural households during social interactions, often closely linked to social relationships, kinship ties, and social status in rural China. The main reason for including this as a control variable is that it reflects the social capital and interpersonal relationship status of farm households, which can influence their economic behavior and poverty status. Detailed definitions and descriptive statistics of these variables are provided in Table 2.

**Table 2:** Descriptive statistics of variables.

	**Variant**	**Define**	**Observed value**	**Average value**	**Standard deviation**
Dependent variables	Multidimensional relative poverty index	Calculated based on the multidimensional relative poverty index system in Table 1	5,303	0.289	0.216
	Income capability poverty index		5,303	0.104	0.154
	Development capability poverty index		5,303	0.135	0.090
	Living environment poverty index		5,303	0.050	0.057
Core independent variables	Formal financial participation	Rural households that have obtained loans from formal financial institutions or maintain bank deposits are designated a value of 1, otherwise 0	5,303	0.301	0.459
	Informal financial participation	Rural households that receive loans from other organizations or individuals, or provide loans to them, are assigned a value of 1; otherwise 0	5,303	0.308	0.462
Head of household control variable	Gender of household head	Sex of the household head (1 = male, 0 = female)	5,303	0.517	0.500
	Age of head of household	Age of the household head: continuous variable (years)	5,303	50.933	11.435
	Age of head of household squared/100	Age of the head of household squared divided by 100	5,303	27.249	11.247
	Political affiliation of the head of household	Whether the head of household is a party member (1 = party member, 0 = mass, democratic party)	5,303	0.006	0.074
	Marriage of head of household	Marital status of household head (1 = married or cohabiting, 0 = unmarried, divorced or widowed)	5,303	0.889	0.314
Household control variable	Household size	Number of household members (persons)	5,303	4.119	1.917
	Per capita savings	Amount of cash and savings per capita in the household (yuan)	5,303	9,525.926	23,306.842
	Per capita expenditure on favors	Total expenditure on gifts and gratuities per capita in the household (yuan)	5,303	1,187.091	1,893.292
Social control variables	Government Subsidies	Whether receiving government subsidies (1 = yes, 0 = no)	5,303	0.598	0.449
	Social Donation	Whether received social donations (1 = yes, 0 = no)	5,303	0.016	0.126

Table 3 categorizes rural households based on their engagement in formal and informal financial activities and compares the mean values of multidimensional relative poverty indices among different subgroups, without accounting for the influence of other variables. Comparison between subgroup (1) and subgroup (2) indicates that the multidimensional relative poverty index of rural households engaged in formal finance is 0.077 lower than that of rural households not engaged in formal finance. Similarly, comparison between subgroup (1) and subgroup (3) reveals that the multidimensional relative poverty index of rural households participating in informal finance is 0.045 lower than that of those not participating in informal finance. The coefficients of comparison demonstrate that the poverty reduction effect of formal financial participation is superior to that of informal finance. A comparison between subgroup (2) and subgroup (4) indicates that farmers participating in both formal and informal finance have a lower mean value of the multidimensional relative poverty index compared to those involved solely in formal finance. This suggests that informal financial participation may continue to complement formal financial engagement in reducing poverty, as evidenced by the decrease in multidimensional relative poverty among rural households. Empirical tests are necessary to verify this observation.

**Table 3:** Comparison of subgroups.

**Subgroups**	**Formal financial participation**	**Informal financial participation**	**Number of observations**	**Percentage of observations**	**Mean multidimensional relative poverty index**	**Difference between groups from (1)**
(1)	No	No	2,616	49.33%	0.315	0
(2)	No	No	1,053	19.86%	0.238	−0.077^***^
(3)	No	No	1,089	20.52%	0.270	−0.045^***^
(4)	No	No	545	10.28%	0.188	−0.127^***^

^***^indicates significant at the 1% level.

### 4.3 Model specification

Firstly, the study assesses the influence of formal and informal financial engagement by rural households on multidimensional relative poverty through a multiple linear regression model, structured as follows:


(6) $$\begin{array}{l} \text{poorinde}{\text{x}}_{\text{i}}=\alpha +\beta_{1}\text{zgj}{\text{r}}_{\text{i}}+\beta_{2}\text{fzgj}{\text{r}}_{\text{i}}+\sum \gamma_{j}\text{contro}{\text{l}}_{\text{ij}}+\varepsilon_{\text{i}} \end{array}$$

In the equation, “poorindex_i_” represents the multidimensional relative poverty index of household “*i*”; “*zgjr*_*i*_” and “*fzgjr*_*i*_” represent the dummy variables for formal and informal financial participation of household “*i*, ” respectively; “*control*_*ij*_” represents the control variables, with per capita savings and per capita expenditure on favors and gifts logarithmically transformed to address potential heteroskedasticity issues; “α_1_”, “α_2_, ” and “β_*j*_” are the parameters to be estimated, and “ε_*i*_” is the random error term. Meanwhile, this study substitutes the multidimensional relative poverty index in the independent variables of Equation 6 with poverty indices representing the three dimensions: income capacity, development capacity, and living environment. This substitution allows for the assessment of rural households' impacts on various dimensions of poverty.

Secondly, the multiple linear regression model computes coefficient sizes from an average standpoint and does not capture the varying impact of formal and informal financial participation on rural households with different levels of multidimensional poverty. Therefore, the study establishes the following quantile regression model:


(7) $$\begin{array}{r} poorindex_{i,q}=\alpha +\varphi_{1i,q}zgjr_{i}+\varphi_{2i,q}fzgjr_{i}\\ +\sum \gamma_{j,q}control_{ij}+\varepsilon_{i} \end{array}$$

In this equation, “*q*” represents the quantile point. This study selects five quantiles of the multidimensional relative poverty index, ranging from small to large, specifically 10%, 25%, 50%, 75%, and 90%. The core and control variables remain consistent with those in Equation 6, where “φ_1*i*_”, “φ_2*i*_, ” and “γ_*j*_” represent the coefficients to be estimated, and “ε_*i*_” signifies the random error term.

## 5 Results and discussions

### 5.1 Results of the baseline regression

This study examines the impact of formal and informal financial participation among rural households on multidimensional relative poverty, as outlined in Equation 6. The regression results for Equation 1 in Table 4 indicate that the estimated coefficient for formal financial participation is −0.025, suggesting that, controlling for other variables, farmers' multidimensional relative poverty index decreases by an average of 0.025 units with formal financial participation. As this negative coefficient is statistically significant at the 1% level, it indicates a strong association between formal financial participation and the reduction of multidimensional relative poverty. While formal finance contributes to reducing poverty among rural households, the effect size remains relatively modest, reflecting the constraints of rural financial development. Thus, expanding formal financial services and increasing financial support could further mitigate multidimensional relative poverty. Similarly, the regression results for Equation 2 show that the estimated coefficient for informal financial participation is −0.014, indicating a 0.014-unit reduction in the multidimensional relative poverty index for farmers engaged in informal finance, controlling for other variables. This coefficient is statistically significant at the 5% level, demonstrating that under certain conditions, informal financial participation also contributes to reducing poverty among rural households. By comparing the coefficients from Equations 1, 2, it is evident that formal financial participation has a more pronounced effect in reducing multidimensional relative poverty compared to informal financial participation. Equation 3 includes both formal and informal financial participation in the model, and after conducting the correlation test, the correlation coefficient between the two is found to be 0.047, well below the critical value of 0.8. This indicates that the correlation between formal and informal financial participation is very low, and there is no significant issue of multicollinearity. Therefore, the regression model can accurately assess the independent impacts of formal and informal financial participation on multidimensional relative poverty, supporting the validity of the model and ensuring the accuracy and robustness of the regression estimates. The results of Equation 3 show that both types of financial participation have a significant negative effect on multidimensional relative poverty among farm households, confirming hypothesis 1.

**Table 4:** The influence of formal and informal financial participation on multidimensional relative poverty among rural households.

**Variant**	**Dependent variable: Multidimensional relative poverty index**		
	**Equation 1**	**Equation 2**	**Equation 3**
Formal financial participation	−0.025^***^ (0.006)		−0.024^***^ (0.006)
Informal financial participation		−0.014^**^ (0.006)	−0.012^**^ (0.006)
Gender	−0.001 (0.006)	−0.002 (0.006)	−0.001 (0.006)
Age	−0.0100^***^ (0.002)	−0.0100^***^ (0.002)	−0.0100^***^ (0.002)
Age squared	0.012^***^ (0.002)	0.012^***^ (0.002)	0.012^***^ (0.002)
Political affiliation	−0.002 (0.037)	−0.002 (0.037)	−0.001 (0.037)
Marriage	−0.011 (0.009)	−0.012 (0.009)	−0.011 (0.009)
Household Size	0.020^***^ (0.002)	0.020^***^ (0.002)	0.020^***^ (0.002)
Per capita savings	−0.011^***^ (0.001)	−0.012^***^ (0.001)	−0.011^***^ (0.001)
Per capita expenditure on favors	0.017^***^ (0.001)	0.017^***^ (0.001)	0.017^***^ (0.001)
Government subsidies	−0.050^***^ (0.006)	−0.050^***^ (0.006)	−0.050^***^ (0.006)
Social donation	−0.062^***^ (0.022)	−0.063^***^ (0.022)	−0.063^***^ (0.022)
Constant term	0.568^***^ (0.043)	0.568^***^ (0.043)	0.570^***^ (0.043)
*R* ^2^	0.159	0.158	0.160
*N*	5,303	5,303	5,303

^***^ and ^**^ indicate significant at the 1% and 5% levels, respectively.

The estimated coefficients of the control variables in Equation 3 indicate that the coefficient for age is −0.010, while the coefficient for age squared is 0.012; both are significant at the 1% level. This suggests that the age of the household head has an inverted U-shaped relationship with the multidimensional relative poverty index: younger and older heads of households are at greater risk of poverty, while middle-aged heads face a lower risk. This relationship arises because middle-aged household heads typically have more opportunities for labor market participation and lower levels of multidimensional relative poverty. In contrast, younger household heads tend to have less work experience and lower wages, while older household heads may encounter health problems or declining incomes, resulting in higher levels of multidimensional relative poverty. The estimated coefficient of household size is 0.020 and is significant at the 1% level, indicating that for each additional household member, the multidimensional relative poverty index increases significantly by 0.020 units. This occurs because household size reflects the number of members within the household; more family members increase the pressure on resource allocation, which may reduce the resources available to each member and heighten the likelihood of multidimensional relative poverty. The estimated coefficient of per capita savings is −0.011, significant at the 1% level, indicating that a 1% increase in per capita savings would significantly reduce the multidimensional relative poverty index by 0.011%. This suggests that per capita savings reflect a household's wealth accumulation, serving as a safeguard against emergencies, medical expenses, or educational investments; thus, higher per capita savings are generally associated with a lower risk of poverty. The estimated coefficient for per capita expenditure on favors is 0.017, also significant at the 1% level, indicating that a 1% increase in per capita expenditure on favors will significantly raise the multidimensional relative poverty index by 0.017%. This is primarily because expenditure on favors often imposes a considerable burden in rural societies, particularly for low-income households, thereby increasing their risk of poverty. The estimated coefficient for government subsidies is −0.050, significant at the 1% level, suggesting that households receiving government subsidies experience a significant reduction of 0.050 units in the multidimensional relative poverty index. This occurs because government subsidies provide direct financial support, alleviating financial burdens related to daily living, medical care, and agricultural production, thus reducing the risk of multidimensional relative poverty. The estimated coefficient for social donations is −0.063, also significant at the 1% level, indicating that households receiving social donations experience a significant reduction of 0.063 units in their multidimensional relative poverty index. This is because social donations offer financial assistance during critical hardships, mitigating poverty risk. The effects of the head of household's gender, marital status, and political affiliation did not pass the significance test, indicating that these factors do not significantly affect the multidimensional relative poverty index.

After substituting the independent variables with the three-dimensional poverty indices, the regression results presented in Table 5 demonstrate that both formal and informal financial participation can exert a detrimental impact on the three dimensions of poverty among rural households. Furthermore, financial participation has varying impacts on different dimensions of poverty, as reflected by the absolute values of the coefficients. Specifically, the coefficients for formal financial participation's effect on the income capacity poverty index, development capacity poverty index, and living environment poverty index are −0.014, −0.008, and −0.006, respectively. This means that on average, farmers who participate in formal finance experience a reduction of 0.014 units in the income capacity poverty index, 0.008 units in the development capacity poverty index, and 0.006 units in the living environment poverty index. Formal financial participation thus has the greatest negative impact on the income capacity poverty dimension, followed by the development capacity poverty dimension, and the smallest impact on the living environment poverty dimension. Similarly, the coefficients for informal financial participation's effect on the income capacity poverty index, development capacity poverty index, and living environment poverty index are −0.013, −0.006, and −0.004, respectively. This indicates that farmers participating in informal finance experience an average reduction of 0.013 units in the income capacity poverty index, 0.006 units in the development capacity poverty index, and 0.004 units in the living environment poverty index. Informal financial participation also has the largest negative impact on the income capacity poverty dimension, followed by the development capacity poverty dimension, and the smallest impact on the living environment poverty dimension.

**Table 5:** Impact of formal and informal financial participation on three dimensions of poverty among rural households.

**Variant**	**Income capability poverty index**	**Development capability poverty index**	**Living environment poverty index**
	**Equation 1**	**Equation 2**	**Equation 3**
Formal financial participation	−0.014^***^ (0.005)	−0.008^***^ (0.003)	−0.006^***^ (0.002)
Informal financial participation	−0.013^***^ (0.004)	0.006^***^ (0.002)	−0.004^***^ (0.001)
Control variable	Controlled	Controlled	Controlled
Constant term	0.258^***^ (0.032)	0.192^***^ (0.019)	0.120^***^ (0.012)
*R* ^2^	0.116	0.113	0.065
*N*	5,303	5,303	5,303

^***^ indicates significant at the 1% level; control variables are as in Table 4.

### 5.2 Endogeneity and robustness tests

#### 5.2.1 Endogeneity test

This study may face a mutually causal endogeneity issue. That is, as the level of multidimensional relative poverty increases for a rural household, financial institutions become more hesitant to provide financial services, making it harder for the rural household to engage in financial activities, and the absence of financial participation exacerbates the multidimensional relative poverty experienced by the rural household. Therefore, this study draws on the research approaches of Dong et al. and He et al., employing the instrumental variable method to address potential endogeneity issues (47, 48). Firstly, rural households are categorized based on county and age. Counties are grouped according to their county codes, while age groups include [18, 30), [30, 40), [40, 50), [50, 60), and [60, 70]. Subsequently, rural households within the same age group and county are grouped together, and the average level of formal financial participation among these households is computed as the instrumental variable for formal financial participation. Similarly, the average level of informal financial participation among rural households within each group serves as the instrumental variable for informal financial participation. The rationale for selecting this instrumental variable is as follows: (1) Rural areas represent traditional communities where interpersonal connections play a significant role, and the financial decisions of one rural household can influence others. Thus, the financial participation of neighboring rural households can impact whether the surveyed households choose formal or informal finance, meeting the relevance criterion for instrumental variable selection. (2) The average level of formal or informal financial participation among neighboring rural households does not directly impact the multidimensional relative poverty of the surveyed rural households. Moreover, the financial engagement of other rural households is beyond the control of the surveyed households, fulfilling the exogeneity criterion for selecting instrumental variables.

In this study, the two-stage least squares (2SLS) method is employed, and the estimation outcomes are presented in Table 6. In the initial stage, the regression coefficients of the instrumental variables are 0.880 and 0.963, respectively, both passing the 1% significance threshold. This demonstrates that the chosen instrumental variables meet the correlation criteria. Additionally, a weak instrumental variable test is performed in this study, yielding a Wald F statistic of 387.95. This result suggests the absence of a weak instrumental variable problem and confirms the effective control of endogeneity. In the second stage, accounting for endogeneity, the coefficients of formal and informal financial participation remain statistically significant at the 1% level. This consistency with earlier findings underscores the robustness of the results.

**Table 6:** Endogeneity treatment: 2SLS instrumental variables approach.

**Variant**	**Phase I**			**Phase II**
	**Formal financial participation**	**Informal financial participation**		**Multidimensional relative poverty index**
Instrumental variable: average formal financial participation level within same age group and county	0.880^***^ (0.025)		Formal financial participation	−0.102^***^ (0.016)
Instrumental variable: average informal financial participation level within same age group and county		0.963^***^ (0.029)	Informal financial participation	−0.060^***^ (0.016)
Control variable	Controlled	Controlled	Control variable	Controlled
Instrumental variable *t*-value	35.20	33.21		
One-stage F-value	613.36	566.19		
Weak instrumental variable test: Wald *F* statistic	387.95			
*R* ^2^	0.268	0.167		0.163
*N*	5,303	5,303	*N*	5,303

^***^indicates significant at the 1% level; control variables are as in Table 4.

Furthermore, the coefficients for formal and informal financial participation in Table 6 are −0.102 and −0.060, respectively, reflecting a significant change from the coefficients of −0.025 and −0.014 in Table 4, which did not account for endogeneity. This alteration primarily results from the endogeneity treatment applied in Table 6 using a pragmatic instrumental variables approach, which mitigates potential self-selection bias and the influence of other unobserved factors, leading to more accurate estimates of causality. Specifically, the coefficient for formal financial participation in Table 6 increases to −0.102, indicating that its poverty reduction effect is more pronounced than suggested in Table 4, which underestimates the role of formal financial participation. Similarly, the coefficient for informal financial participation rises from −0.014 in Table 4 to −0.060 in Table 6, implying that the effect of informal financial participation on poverty reduction becomes more significant after addressing endogeneity. These findings indicate that the poverty reduction effect of financial participation is underestimated when endogeneity is not addressed. With the endogeneity treatment, the regression results in Table 6 reveal a stronger impact of financial participation on multidimensional relative poverty. This suggests that policymakers should not only promote the development of formal finance but also recognize the essential complementary role of informal finance in poverty alleviation.

#### 5.2.2 Robustness test

This study employs the propensity score matching method to conduct robustness testing. Formal or informal financial participation faces a self-selection issue, wherein rural households make deliberate choices based on their resources, financial status, and employment orientation. This non-random selection violates the principle of random sampling. Thus, this study employs the propensity score matching (PSM) method to address potential selectivity bias. To assess the poverty alleviation impact of formal financial participation on multidimensional relative poverty and whether informal financial participation can sustain its poverty reduction role, this study conducts two propensity score matching (PSM) estimations. The first estimation comprises a total of 5,303 rural households, with 1,598 households participating in formal finance as the experimental group, and 3,705 households not participating in formal finance as the control group. For the second estimation, the sample consists of 1,598 households engaged in formal finance, of which 545 households are also participating in informal finance as the experimental group, while 1,053 households not participating in informal finance serve as the control group. Table 7 shows that the average treatment effect (ATT) in both propensity score matching (PSM) estimations passes the 1% significance test, except for the second estimation where the impact of informal financial participation on the multidimensional relative poverty index in nearest neighbor matching only meets the 5% significance test. The negative and relatively small ATT values across different matching methods confirm the effectiveness of financial participation in reducing multidimensional relative poverty. The poverty reduction effect remains significant and consistent regardless of the matching method used, indicating the robustness of the results and further supporting the effectiveness of financial participation. This outcome reaffirms the robustness and validity of the findings, indicating that both formal and informal financial participation among rural households contribute to the reduction of multidimensional relative poverty.

**Table 7:** Robustness test: propensity score matching method.

			**Multidimensional relative poverty index**	**Income capability poverty index**	**Development capability poverty index**	**Living environment poverty index**
**Full sample**, ***N*** = **5,303**						
Formal financial participation	Nearest neighbor matching	ATT	−0.027^***^	−0.014^***^	−0.009^***^	−0.006^***^
	Radius matching	ATT	−0.022^***^	−0.014^***^	−0.009^***^	−0.005^***^
	Kernel matching	ATT	−0.023^***^	−0.015^***^	−0.009^***^	−0.006^***^
**Rural households with formal financial participation**, ***N*** = **1,598**						
Informal financial participation	Nearest neighbor matching	ATT	−0.013^**^	−0.015^***^	−0.006^***^	−0.004^***^
	Radius matching	ATT	−0.012^***^	−0.014^***^	−0.006^***^	−0.004^***^
	Kernel matching	ATT	−0.012^***^	−0.015^***^	−0.006^***^	−0.004^***^

^***^ and ^**^ indicate significant at the 1% and 5% levels, respectively.

### 5.3 Mechanism analysis

This section employs a mediation effect model to delve deeper into the mechanism through which the formal and informal financial engagement of rural households influences multidimensional relative poverty. Drawing from preceding analyses, land transfer and non-farm employment are chosen as mediating variables for examination. Among rural households, land transfer encompasses both the acquisition and relinquishment of land. If a household leases out land allocated by the collective or rents land from individuals or the collective, it is designated as engaging in land transfer and assigned a value of 1; otherwise, it is assigned a value of 0. Non-farm employment among rural households encompasses both entrepreneurial activities and work conducted outside the home. If any member of the household is involved in self-employment, establishes a business, or commutes for work, the household is classified as engaged in non-farm employment and is assigned a value of 1; otherwise, it is assigned a value of 0.

Table 8 presents the estimation results of the mediation effects model of land transfers. Equations 1–3 are employed to examine the mechanism by which formal financial participation of farm households affects multidimensional relative poverty, with land transfer serving as the mediating variable. According to the estimation results of Equation 2, the coefficient of formal financial participation is 0.038 and is significant at the 1% level, indicating that farmers' participation in formal finance increases the likelihood of land transfer by 3.8%. From the results of Equation 3, the estimated coefficient of land transfer is −0.021, also significant at the 1% level, suggesting that land transfer decreases the multidimensional relative poverty index of farmers by 2.1%. Compared to the coefficient of formal financial participation in Equation 1, which is −0.025, the coefficient in Equation 3 decreases slightly to −0.024, showing that the negative effect on multidimensional relative poverty weakens. This indicates the presence of a partial mediation effect, where formal financial participation promotes land transfer, which in turn helps reduce multidimensional relative poverty. Similarly, Equations 4–6 are employed to examine the mechanism through which informal financial participation of farm households affects multidimensional relative poverty, with land transfer acting as a mediating variable. According to the estimation results of Equation 5, the coefficient of informal financial participation is 0.079, significant at the 1% level, indicating that farmers' participation in informal finance increases the likelihood of land transfer by 7.9%. From the results of Equation 6, the estimated coefficient of land transfer is −0.021, also significant at the 1% level, suggesting that land transfer decreases the multidimensional relative poverty index of farm households by 2.1%. A comparison with the coefficient of −0.014 for informal financial participation in Equation 4 shows that the coefficient in Equation 6 decreases slightly to −0.012, indicating that the negative effect on multidimensional relative poverty weakens. This suggests the presence of a partial mediation effect, where informal financial participation facilitates land transfer, thereby reducing multidimensional relative poverty. Additionally, the comparison reveals that while both formal and informal financial participation reduce multidimensional relative poverty through land transfer, the mediating effect of informal financial participation plays a more significant role in this process.

**Table 8:** Mechanism test: mediating effect model of land transfers.

**Modeling the mediating effects of land transfers**							
**Variant**	**Equation 1**	**Equation 2**	**Equation 3**	**Variant**	**Equation 4**	**Equation 5**	**Equation 6**
	**Multidimensional relative poverty index**	**Land transfer**	**Multidimensional relative poverty index**		**Multidimensional relative poverty index**	**Land transfer**	**Multidimensional relative poverty index**
Formal financial participation	−0.025^***^ (0.006)	0.038^***^ (0.014)	−0.024^***^ (0.006)	Informal financial participation	−0.014^**^ (0.006)	0.079^***^ (0.013)	−0.012^**^ (0.006)
Land transfer			−0.021^***^ (0.006)	Land transfer			−0.021^***^ (0.006)
Control variable	Controlled	Controlled	Controlled	Control variable	Controlled	Controlled	Controlled
Constant term	0.568^***^ (0.043)	−0.046 (0.095)	0.567^***^ (0.043)	Constant term	0.568^***^ (0.043)	−0.058 (0.095)	0.566^***^ (0.043)
R^2^	0.159	0.123	0.161	R^2^	0.158	0.176	0.159
*N*	5,303	5,303	5,303	*N*	5,303	5,303	5,303

^***^ and ^**^ indicate significant at the 1% and 5% levels, respectively; control variables are the same as in Table 4.

Table 9 presents the estimation results of the mediation effects model of non-farm employment. Equations 1 through 3 examine the mechanism by which formal financial participation of farm households influences multidimensional relative poverty, with non-farm employment as the mediating variable. According to the estimation results of Equation 2, the coefficient of formal financial participation is 0.035, significant at the 1% level, indicating that participation in formal finance increases the probability of non-farm employment by 3.5%. From the results of Equation 3, the estimated coefficient of non-farm employment is −0.079, also significant at the 1% level, suggesting that non-farm employment reduces the multidimensional relative poverty index of farm households by 7.9%. Comparing this to the coefficient of formal financial participation in Equation 1, which is −0.025, shows that the coefficient in Equation 3 decreases slightly to −0.022, weakening the negative effect on multidimensional relative poverty. This indicates the presence of a partial mediation effect, where formal financial participation plays a key role in promoting non-farm employment, which in turn contributes to reducing multidimensional relative poverty. Similarly, Equations 4 through 6 examine the mechanism by which informal financial participation of farm households affects multidimensional relative poverty, using non-farm employment as the mediating variable. According to the results of Equation 5, the coefficient of informal financial participation is 0.076, significant at the 1% level, indicating that informal financial participation increases the probability of non-farm employment by 7.6%. In Equation 6, the estimated coefficient of non-farm employment is −0.078, also significant at the 1% level, showing that non-farm employment reduces the multidimensional relative poverty index of farm households by 7.8%. Comparing the coefficient of informal financial participation in Equation 4, which is −0.014, with that in Equation 6, which decreases to −0.010, suggests a weaker negative effect on multidimensional relative poverty, indicating a partial mediating effect. This suggests that informal financial participation also reduces multidimensional relative poverty by promoting non-farm employment. Moreover, the comparison indicates that while both formal and informal financial participation reduce multidimensional relative poverty through non-farm employment, the mediating effect of informal financial participation plays a larger role. In summary, both forms of financial participation significantly promote land transfer and non-farm employment, thereby reducing multidimensional relative poverty, with informal financial participation having a stronger mediating effect in both processes. Hypothesis 2 is examined.

**Table 9:** Mechanism test: mediating effect model of non-farm employment.

**Modeling the mediating effects of non-farm employment**							
**Variant**	**Equation 1**	**Equation 2**	**Equation 3**	**Variant**	**Equation 4**	**Equation 5**	**Equation 6**
	**Multidimensional relative poverty index**	**Non-farm employment**	**Multidimensional relative poverty index**		**Multidimensional relative poverty index**	**Non-farm employment**	**Multidimensional relative poverty index**
Formal financial participation	−0.025^***^ (0.006)	0.035^***^ (0.012)	−0.022^***^ (0.006)	Informal financial participation	−0.014^**^ (0.006)	0.076^***^ (0.014)	−0.010^***^ (0.003)
Non-farm employment			−0.079^***^ (0.006)	Non–farm employment			−0.078^***^ (0.006)
Control variable	Controlled	Controlled	Controlled	Control variable	Controlled	Controlled	Controlled
Constant term	0.568^***^ (0.043)	−0.024 (0.102)	0.566^***^ (0.042)	Constant term	0.568^***^ (0.043)	−0.039 (0.101)	0.565^***^ (0.042)
R^2^	0.159	0.110	0.188	R^2^	0.158	0.115	0.186
*N*	5,303	5,303	5,303	N	5,303	5,303	5,303

^***^ and ^**^ indicate significant at the 1% and 5% levels, respectively; control variables are the same as in Table 4.

### 5.4 Heterogeneity analysis

Table 10 displays the results based on regression quartiles. The findings indicate that as the quantile point increases, the coefficient of formal financial participation decreases while the coefficient of informal financial participation increases. This suggests that formal financial participation has a more pronounced impact on poverty reduction among rural households with lower levels of multidimensional relative poverty, whereas informal financial participation plays a more significant role in poverty reduction among rural households facing higher levels of multidimensional relative poverty. Specifically, the negative impact of formal financial participation by farmers on multidimensional relative poverty diminishes from 0.043 at the 10th quantile to 0.006 at the 90th quantile. This suggests that formal financial participation is more effective in alleviating poverty among farmers with lower levels of multidimensional relative poverty. Lower-poverty-level farmers can leverage the advantages of formal finance—such as larger loan sizes and lower costs—to make productive investments, resulting in higher economic returns and reduced poverty risk. In contrast, informal financial participation has a non-significant effect on the multidimensional relative poverty of farmers at the 10th and 25th quantiles but shows a significant negative effect starting from the 50th quantile, increasing from 0.009 to 0.021 at the 90th quantile. This indicates that informal financial participation is more effective in alleviating poverty for farmers with higher levels of multidimensional relative poverty. For these farmers, informal finance provides a crucial source of short-term funds that helps them manage daily financial shortages due to limited access to formal financial support. Thus, the poverty reduction effect of informal finance is particularly significant for this group. These results validate Hypothesis 3 and indicate that poverty alleviation strategies should fully leverage the advantages of both formal and informal finance while utilizing their complementary roles.

**Table 10:** Heterogeneity analysis: regression quartiles.

**Variant**	**Dependent variable: Multidimensional relative poverty index**				
	**Q10**	**Q25**	**50**	**Q75**	**Q90**
Formal financial participation	−0.043^***^ (0.012)	−0.036^***^ (0.010)	−0.019^***^ (0.007)	−0.016^**^ (0.008)	−0.006^**^ (0.003)
Informal financial participation	0.001 (0.005)	0.002 (0.006)	−0.009^**^ (0.045)	−0.020^***^ (0.006)	−0.021^***^ (0.007)
Control variable	Controlled	Controlled	Controlled	Controlled	Controlled
Constant term	0.233^***^ (0.037)	0.345^***^ (0.037)	0.605^***^ (0.061)	0.757^***^ (0.008)	0.817^***^ (0.088)
*N*	5,303	5,303	5,303	5,303	5,303

^***^ and ^**^ indicate significance at the 1% and 5% levels, respectively; control variables remain consistent with those listed in Table 4; q represents the quartile point, with five quartiles −10%, 25%, 50%, 75%, and 90%—selected in ascending order.

Although the quantile regression results in Table 10 indicate that informal finance significantly reduces multidimensional poverty among relatively poorer farm households, this effect is not without risks. Due to its reliance on personal relationships and social networks, informal finance can create overdependence on limited financial sources, hindering long-term economic sustainability. Moreover, the absence of effective regulation may result in high interest rates or unfair lending practices, exacerbating financial burdens on farm households. In regions with uneven resource distribution or weak social capital, informal finance may further deepen inequalities or even trigger debt risks. Therefore, while informal finance plays a crucial complementary role in poverty reduction, its limitations warrant careful attention. Policies aimed at optimizing informal financial services, such as standardizing management practices and developing rural credit systems, are recommended to ensure sustainability and equity.

## 6 Conclusions, managerial implications and limitations

### 6.1 Conclusions

After the elimination of absolute poverty, the promotion of common prosperity should focus on the relative gap, and it is worthwhile to explore whether the formal and informal financial participation of rural households can play a synergistic role in reducing multidimensional relative poverty and ultimately help realize common prosperity. Building upon this foundation, the study utilizes data from the 2018 China Family Panel Studies to establish a multidimensional relative poverty indicator system. It employs a comprehensive analytical framework and integrates both formal and informal financial participation of rural households into the scope of investigation from the demand side. Furthermore, the study empirically examines the mechanisms in addressing multidimensional relative poverty. The study's findings demonstrate that, firstly, both formal and informal financial engagement among rural households can mitigate multidimensional relative poverty, and the poverty alleviation impact of formal financial participation surpasses that of informal financial participation. When multidimensional relative poverty is disaggregated into three dimensions—income capability poverty, developmental capability poverty, and living environment poverty—both types of financial engagement exhibit the most substantial negative effect on income capability poverty, followed by developmental capability poverty, and least on living environment poverty. Secondly, the mechanism analysis reveals that both formal and informal financial engagement of rural households play a crucial role in fostering land transfer and non-farm employment, consequently contributing to the reduction of multidimensional relative poverty. Thirdly, the analysis of heterogeneity indicates that formal financial participation is more efficacious in alleviating poverty among rural households experiencing lower levels of multidimensional relative poverty. Conversely, informal financial participation demonstrates greater effectiveness in poverty reduction for rural households facing higher levels of multidimensional relative poverty. This suggests a complementary relationship between formal and informal finance, emphasizing the necessity of leveraging both formal and informal financial instruments to address financial poverty.

### 6.2 Managerial implications

Based on the aforementioned findings, this study delineates the following policy implications: firstly, enhancing formal finance to leverage the advantages of informal finance. Formal financial institutions can leverage the operational modalities and information accessibility advantages of informal financial institutions. For instance, they can adopt the flexible operational approach of informal finance to offer a broader array of loan products, including microfinance, agricultural production loans, and consumer loans, catering to diverse needs of rural households. Additionally, they can capitalize on the accessibility convenience of informal finance to collaborate with rural cooperatives, mutual aid organizations, or local communities in advancing the development and dissemination of financial products, thereby enhancing outreach and service provision to rural households. This approach fosters the adaptability and inclusivity of financial services, thereby bolstering efforts in multidimensional relative poverty reduction and fostering shared prosperity. Secondly, there should be an increase in financial assistance for land transfer and non-farm employment. Regarding land transfer, the government can not only initiate specialized loan programs to offer financial aid but also establish risk mitigation mechanisms such as land transfer insurance and guarantee insurance. These measures aim to provide a certain level of risk protection for rural households engaged in land transfer, thus alleviating the risk burden they face. Concerning non-agricultural employment, the government can establish dedicated entrepreneurial financing initiatives, such as business loans and venture capital funds, to provide financial support and capital infusion for rural households. These efforts aim to foster innovation, entrepreneurship, and stimulate employment growth. Thirdly, it is imperative to implement tailored financial support policies. For rural households experiencing lower levels of multidimensional relative poverty, the government should enhance its assistance for formal financial engagement. By providing formal financial services such as loans, savings, and insurance, it can address issues related to capital scarcity and risk mitigation for rural households, thus fostering sustainable poverty alleviation. For rural households experiencing significant levels of multidimensional relative poverty, the government should regulate informal financial participation. It should also facilitate access to small loans, savings services, technical assistance, and market opportunities through rural cooperatives, microcredit organizations, or mutual funds. This approach aims to mitigate the exclusion of formal financial services in rural areas, alleviate multidimensional relative poverty, and advance the attainment of shared prosperity.

The findings of this study not only offer empirical support for multidimensional poverty governance in rural China but also hold potential for application in other developing regions. Specifically, the proposed synergy between formal and informal finance, along with the poverty reduction pathways of land transfer and off-farm employment, has broad relevance for agrarian economies characterized by multidimensional poverty. For instance, regions such as South Asia, Southeast Asia, and Sub-Saharan Africa share challenges like financial exclusion, inefficient land use, and weak social protection, making the findings applicable in these contexts. Expanding formal financial coverage through digital financial technology while regulating informal finance can address deficiencies in rural financial services effectively. Additionally, optimizing land transfer policies and promoting non-farm employment can enhance the income and development potential of farm households by improving land market mechanisms and offering vocational training. The multidimensional relative poverty monitoring approach proposed in this study also provides a framework for constructing scientific poverty assessment systems in other countries. However, adaptation challenges may arise due to variations in financial market maturity, socio-cultural contexts, and infrastructure levels. Thus, policy adjustments should consider local characteristics, such as introducing government guarantees or international assistance to enhance financial stability and promoting infrastructure development and digitalization to support financial service expansion. By fostering international cooperation and experience sharing, and tailoring policies to specific national conditions, the findings of this study can serve as a reference for rural poverty governance worldwide, contributing to the global pursuit of common prosperity.

### 6.3 Limitations and future research

While this study contrasts the effects of formal and informal financial engagement among rural households on multidimensional relative poverty, yielding significant theoretical and practical insights, it also identifies several limitations that suggest potential avenues for future research. Firstly, cross-sectional data presents certain limitations. This study utilizes data from 2018, yet a one-year timeframe may not fully capture the enduring effects of financial participation on multidimensional relative poverty. For instance, financial participation encompasses borrowing and lending activities, and while short-term borrowing and lending behaviors might alleviate multidimensional relative poverty among rural households, they could potentially exacerbate it in the long term. Future research could explore data spanning longer periods to enable more comprehensive trend analysis and deepen our comprehension of the correlation between financial participation and multidimensional relative poverty. Secondly, infrastructure is absent from the mechanism analysis. Financial participation can contribute to enhancing infrastructure, including housing, water, sanitation, and other facilities, thereby enhancing the living standards of rural households and mitigating multidimensional relative poverty. Future mechanism analyses could incorporate considerations of infrastructure development.

## Data Availability

Publicly available datasets were analyzed in this study. This data can be found here: https://www.isss.pku.edu.cn/cfps/index.htm, China Family Panel Studies.
